# Thioredoxin 1 mediates TGF-β-induced epithelial-mesenchymal transition in salivary adenoid cystic carcinoma

**DOI:** 10.18632/oncotarget.4635

**Published:** 2015-08-17

**Authors:** Yang Jiang, Xin Feng, Lei Zheng, Sheng-Lin Li, Xi-Yuan Ge, Jian-Guo Zhang

**Affiliations:** ^1^ Department of Oral and Maxillofacial Surgery, Peking University School and Hospital of Stomatology, Beijing 100081, PR China; ^2^ Department of Otolaryngology, Wake Forest School of Medicine, Winston-Salem, NC 27157, USA; ^3^ Central Laboratory, Peking University School and Hospital of Stomatology, Beijing 100081, PR China

**Keywords:** thioredoxin 1, epithelial-mesenchymal transition, metastasis, TGF-β, salivary adenoid cystic carcinoma

## Abstract

Epithelial-mesenchymal transition (EMT) plays an important role in the invasion and metastasis of salivary adenoid cystic carcinoma (SACC) which is characterized by wide local infiltration, perineural spread, a propensity to local recurrence and late distant metastasis. Our recent studies have disclosed that TGF-β is a crucial factor for EMT in metastatic SACC. In this study, we further uncovered small redox protein thioredoxin 1 (TXN) as a critical mediator of TGF-β induced EMT. Immunohistochemistry analysis revealed significantly higher expressions of TXN, thioredoxin reductase 1 (TXNRD1) and N-cadherin, and lower expression of E-cadherin in human metastatic SACC compared to non-metastatic SACC tissues. Consistently, cultured SACC cells with stable TXN overexpression had decreased E-cadherin and increased N-cadherin as well as Snail and Slug expressions. The enhanced migration and invasion potential of these cells was abrogated by Akt or TXNRD1 inhibitors. Expression of N-cadherin and Akt p-Akt decreased, whereas E-cadherin expression increased in a BBSKE (TXNRD1 inhibitor)-dose-dependent manner. In a xenograft mouse model, TXN overexpression facilitated the metastatic potential of SACC-83 cells to the lung. Our results indicate that TXN plays a key role in SACC invasion and metastasis through the modulation of TGF-β-Akt/GSK-3β on EMT. TXN could be a potential therapeutic target for SACC.

## INTRODUCTION

Salivary adenoid cystic carcinoma (SACC) is one of the most common malignant tumors of salivary glands that arise from ductal, myoepithelial, and basal cells [1, 2]. Because of persistent indolent growth, high rates of recurrence, and distant metastasis, the overall survival rate is 71% at 5 years, but 37% at 15 years. Survival rates after diagnosis of tumor progression at 5 and 15 years are 35% and 0%, respectively [3]. Surgical dissection and postoperative radiotherapy, the standard treatment, has provided reasonable local control rates but limited response for distant metastasis [4]. A better understanding of the biological mechanisms underlying SACC metastasis is needed to improve this situation.

Epithelial-mesenchymal transition (EMT) is the molecular basis of cancer metastasis [5], and involves loss of cell-cell adhesion, cell polarity, acquisition of migratory and invasive capabilities. In EMT, there are decreased expression of cell adhesion molecules such as E-cadherin and increased expression of mesenchymal markers such as vimentin and N-cadherin [6]. TGF-β, the most potent factor of EMT, has been implicated in various types of cancer cells [7]. Our previous studies have shown that human metastatic SACC tissue samples and SACC-LM cell line had higher expression of TGF-β. In addition, TGF-β-induced EMT promoted the migration and invasion of SACC-83 cells [8, 9]. However, the mechanisms underlying TGF-β-induced EMT in SACC are still unclear, which needs to be investigated.

The thioredoxin system including thioredoxin, thioredoxin reductase and nicotinamide adenine dinucleotide phosphate (NADPH) is involved in aspects of tumor physiology such as proliferation, apoptosis, and metastasis [10]. Thioredoxin 1 (TXN), a 12-kDa redox protein, is important in regulation of cellular redox homeostasis and anti-apoptotic functions. TXN stimulates cell proliferation and cell cycle progression, induces hypoxia-inducible factor-1α (HIF-1α) and angiogenesis, and alters the balance between the matrix metalloproteinases and their tissue inhibitors [11, 12]. Recent studies have shown that TXN had high expression in many human primary tumor tissues such as the liver, colon, pancreas, and the uterine cervix [13–16]. Increased TXN expression may be associated with oncogenesis, a poorer survival rate, and higher reactive oxygen species (ROS) generation [17]. Recent findings have demonstrated that ROS-evoked signals are associated with TGF-β signaling-mediated EMT in breast and renal tubular epithelial cells and in cardiac and pulmonary fibrosis [18–21]. As a negative regulator of TXN, thioredoxin binding protein-2 (TBP-2) deficiency can upregulate the expression of Snail or Slug in TGF-β-driven EMT [22]. However, little is known about the role of TXN in tumor mobility and invasion during the process of TGF-β-induced EMT. We hypothesize that TXN may be involved in the TGF-β-mediated EMT-induced tumor mobility and invasion in SACC.

In this study, we compared the expression of TXN, TXNRD1, E-cadherin and N-cadherin in SACC patients with or without lung metastasis. We also manipulated TXN expression in two SACC cell lines, SACC-83(low lung-metastatic cell line) and SACC-LM (high lung-metastatic cell line) [9] to compare cell-invasive behavior. Silence of TXN decreased SACC-LM cell mobility and invasion, whereas overexpression of TXN increased SACC-83 cell mobility and invasion which was dependent on the Akt/GSK-3β pathway. Our *in vivo* study further found that SACC-83 cells overexpressing TXN had significantly increased potential of lung metastasis. In addition, EMT measured by E-cadherin and N-cadherin and cell invasion was promoted by manipulating TXN expression in SACC-83 cells. These findings suggest that the EMT mediated by TXN and TXNRD1 plays an important role in SACC metastasis. Therefore, TXN and TXNRD1 could be novel targets for SACC treatment in future.

## RESULTS

### Endogenous TXN and TXNRD1 expressions are correlated with the potential of metastasis and poor survival in SACC patients

**Figure 1:**
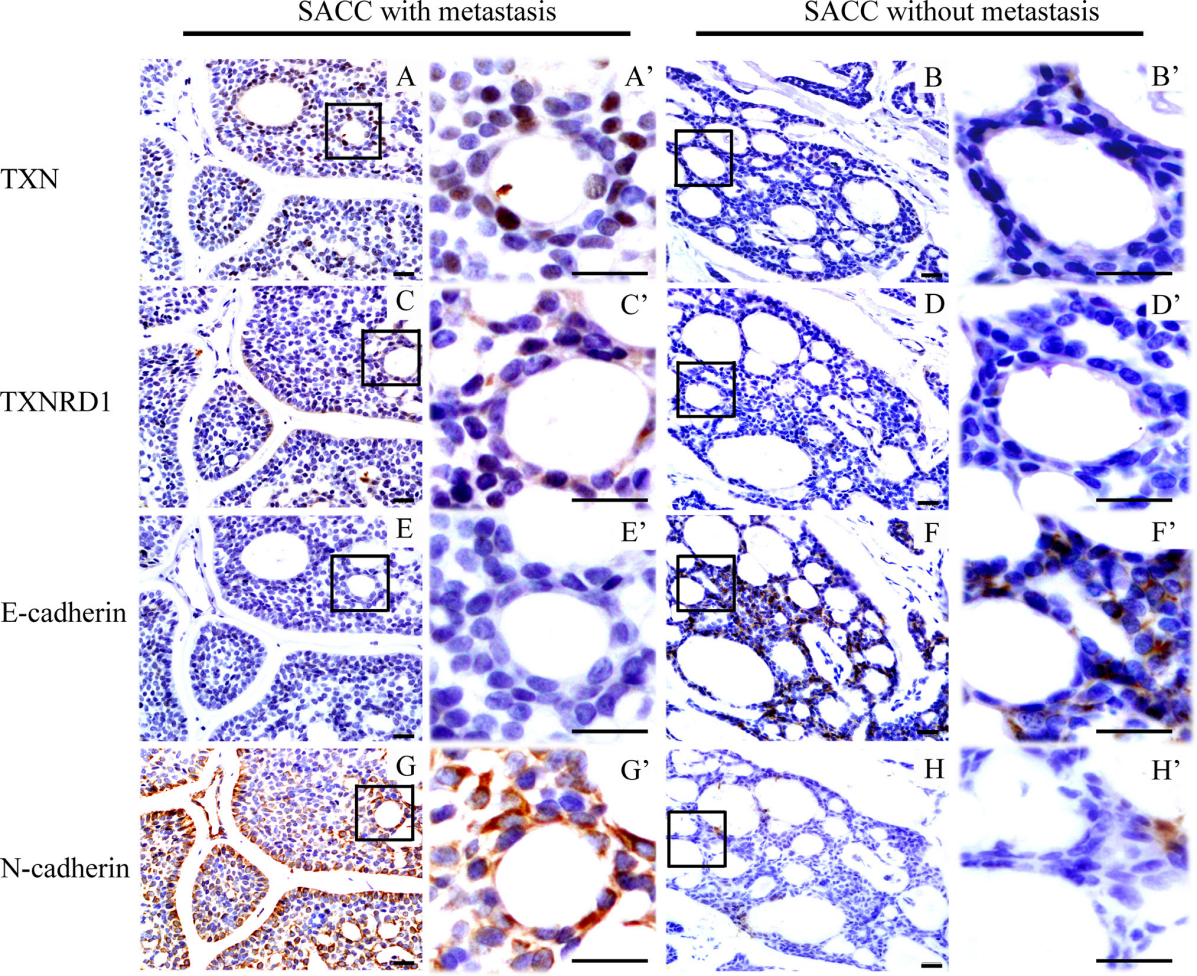
Immunohistochemical staining reveals differential expressions of thioredoxin 1 (TXN), thioredoxin reductase 1 (TXNRD1) and epithelial-mesenchymal transition signs in salivary adenoid cystic carcinoma (SACC) samples from patients with/without lung metastasis Higher expression of TXN (AA’), TXNRD1 (CC’) and N-cadherin (GG’) in SACC tissues with metastasis compared to those without metastasis (BB’, DD’, HH’), respectively. Lower expression of E-cadherin in SACC tissues with metastasis (EE’) compared to those without metastasis (FF’). Scale bar = 20 μm, A’-H’ show enlarged fields of the inset squares in panels **A–H.**

**Figure 2:**
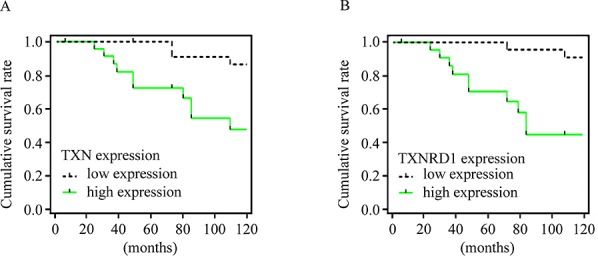
TXN and TXNRD1 expression are correlated with survival rate of patients with SACC Kaplan-Meier survival curves for cumulative survival rate of 47 patients with SACC. Expressions of TXN **A.** and TXNRD1 **B.** were positively correlated with poor survival rate.

Expressions of TXN, TXNRD1, E-cadherin, and N-cadherin were detected by immunohistochemical analysis in SACC tissues with (*n* = 25) or without (*n* = 22) metastasis. SACC tissues with metastasis had high expression of TXN and TXNRD1, which were correlated with high expression of the mesenchymal marker N-cadherin and low expression of the epithelial marker E-cadherin (Figure 1A–1H). Occasional nuclear staining of TXN was also found in the stained tissue sample (Figure 1A’). Kaplan-Meier survival analysis for above 47 SACC patients demonstrated that TXN and TXNRD1 expressions were correlated with poor survival rate (*P* = 0.0072 and *P* = 0.0224, respectively) (Figure 2A and 2B). TXN was expressed in 21 out of 25 SACC samples with metastasis and in 2 out of 22 SACC samples without metastasis (Table 1). Correlations between TXN expression and clinicopathological features of SACC were summarized in Table 1. As shown in Table 1, high TXN expression in SACC was significantly correlated with clinical stage (*P* = 0.012) and distant metastasis (*P* < 0.001). TXN expression in SACC was also positively associated with TXNRD1 (*P* < 0.001), N-cadherin (*P* = 0.018), and negatively associated with E-cadherin (*P* = 0.01) expression (Table 2).

**Table 1:** Thioredoxin 1 (TXN) expression and clinicopathologic features in 47 patients with SACC

	TXN expression level					*p*-value
	total	Low		High		
	*N*	*N*	(%)	*N*	(%)	
**Age (y)**						
< 45	29	14	48.28	15	51.72	0.908
≥ 45	18	9	50	9	50	
**Gender**						
Male	20	8	40	12	60	0.292
Female	27	15	55.56	12	44.44	
**Tumor size**						
T1+T2	31	17	54.84	14	45.16	0.753
T3+T4	16	8	50	8	50	
**Clinical stage**						
I/II	29	19	65.52	10	34.48	0.012*
III/IV	18	5	27.78	13	72.22	
**Lymph node metastasis**						
Absent	44	22	50	22	50	0.578
Present	3	2	66.67	1	33.33	
**Perineural invasion**						
Absent	19	12	63.16	7	36.84	0.172
Present	28	12	42.86	16	57.14	
**Local regional recurrence**						
Absent	13	10	76.92	3	23.08	0.327
Present	34	21	61.76	13	38.24	
**Distant metastasis**						
Absent	22	20	90.91	2	9.10	< 0.001*
Present	25	4	16	21	84	

* Statistically significant

T = Tumor size.

**Table 2:** Relationships among thioredoxin 1 (TXN) expression and thioredoxin reductase (TXNRD1), E-cadherin, and N-cadherin expression

	TXN expression level					*p*-value
	total	Low		High		
	*N*	*N*	(%)	*N*	(%)	
**TXNRD1 expression**						
Low	24	18	75	6	25	< 0.001*
High	23	5	21.74	18	78.26	
**E-cadherin expression**						
Low	31	11	35.48	20	64.52	0.01*
High	16	12	75	4	25	
**N-cadherin expression**						
Low	31	19	61.29	12	38.71	0.018*
High	16	4	25	12	75	

* Statistically significant.

### TXN expression in SACC cell lines potentially impacts on EMT, migration and invasion

**Figure 3:**
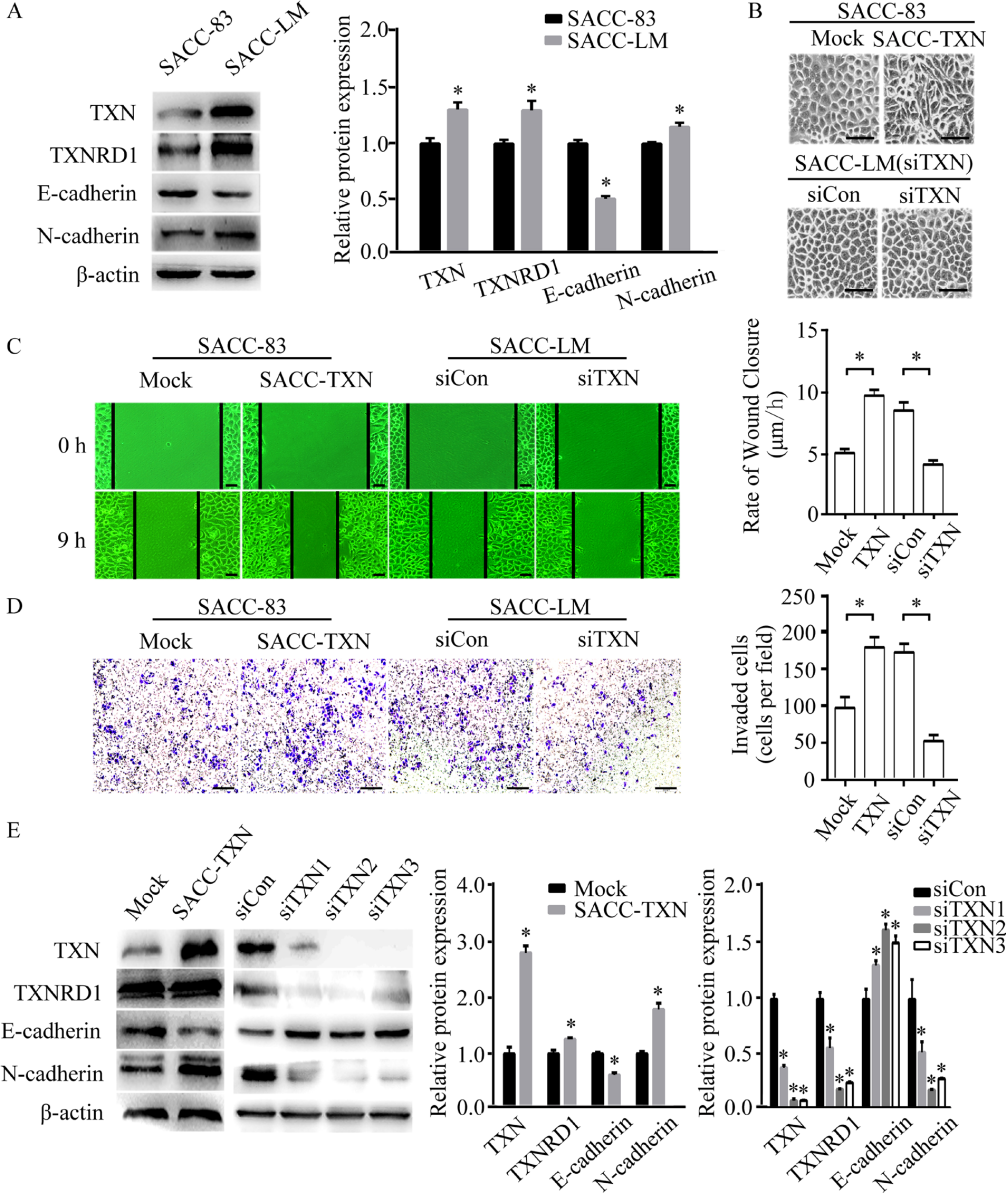
TXN modulates epithelial and mesenchymal proteins and promotes migration and invasion in SACC cell lines **A.** Immunoblotting analysis of TXN, TXNRD1, E-cadherin, and N-cadherin expression in SACC-83 and SACC-LM tumor cell lines. **B.** Phase contrast microscopy revealed fibroblastic morphological changes of epithelial-mesenchymal transition (EMT) in TXN-overexpressed SACC-83 cells (SACC-TXN) compared to control cells (Mock). Knocking down TXN in SACC-LM (siTXN) induced no apparent morphological changes compared with control cells (siCon). Scale bar = 100 μm. **C.** Wound closure assays in Mock, SACC-TXN, siCon, and siTXN cells. Images were captured at 0 and 9 hours after wounding in serum-free media. *N* = 3, scale bar = 50 μm. **D.** Invasion assays (24 hours) for Mock, SACC-TXN, siCon, and siTXN cells. Numbers of invaded cells were compared. *N* = 3, scale bar = 200 μm. **E.** Immunoblotting analysis of TXN, TXNRD1, E-cadherin, and N-cadherin expressions in Mock, SACC-TXN, siCon, and siTXNs (three different TXN targeting siRNAs – siTXN1, siTXN2, and siTXN3) cells. Data represent means ± standard deviation (SD) of three independent experiments (*P < 0.05).

Our previously established SACC-83 and SACC-LM cell lines have identical STR profiling, and express epithelial markers such as pan-cytokeratin and cytokeratin AE1, and the luminal markers such as CK8/18 and S100P, indicating that both cell lines were originated in oral adenoepithelial cells and not contaminated by other cancer cell lines [8]. In this study, we further identified that SACC-83 and SACC-LM cell lines expressed the intrinsic SACC biomarkers including c-myb, FABP7 and NTF3F (Figure S1, see Table S1 for primer sequences). Using these two cell lines, we explored the role of TXN expression in EMT, migration and invasion. Our data showed that SACC-LM cell line had higher expressions of TXN and N-cadherin, but lower expression of E-cadherin compared to SACC-83 cell line (Figure 3A). The overexpression of TXN by transfection of GV230 vector in SACC-83 (SACC-TXN) cells showed a fibroblast-like morphology (Figure 3B). Wound closure and transwell assays revealed that overexpression of TXN increased SACC-83 cell migration and invasion whereas knockdown of TXN decreased SACC-LM cell migration and invasion (Figure 3C and 3D). In addition, TXN overexpression upregulated N-cadherin and downregulated E-cadherin expression in SACC-TXN cells (Figure 3E) whereas TXN knockdown by siRNA in SACC-LM (siTXN) cells upregulated E-cadherin expression and downregulated N-cadherin expression (Figure 3E). These data suggested that TXN may play a potential role in promoting motility, invasion and EMT of SACC cells.

### TXN is a critical mediator in TGF-β-induced EMT in SACC cells

**Figure 4:**
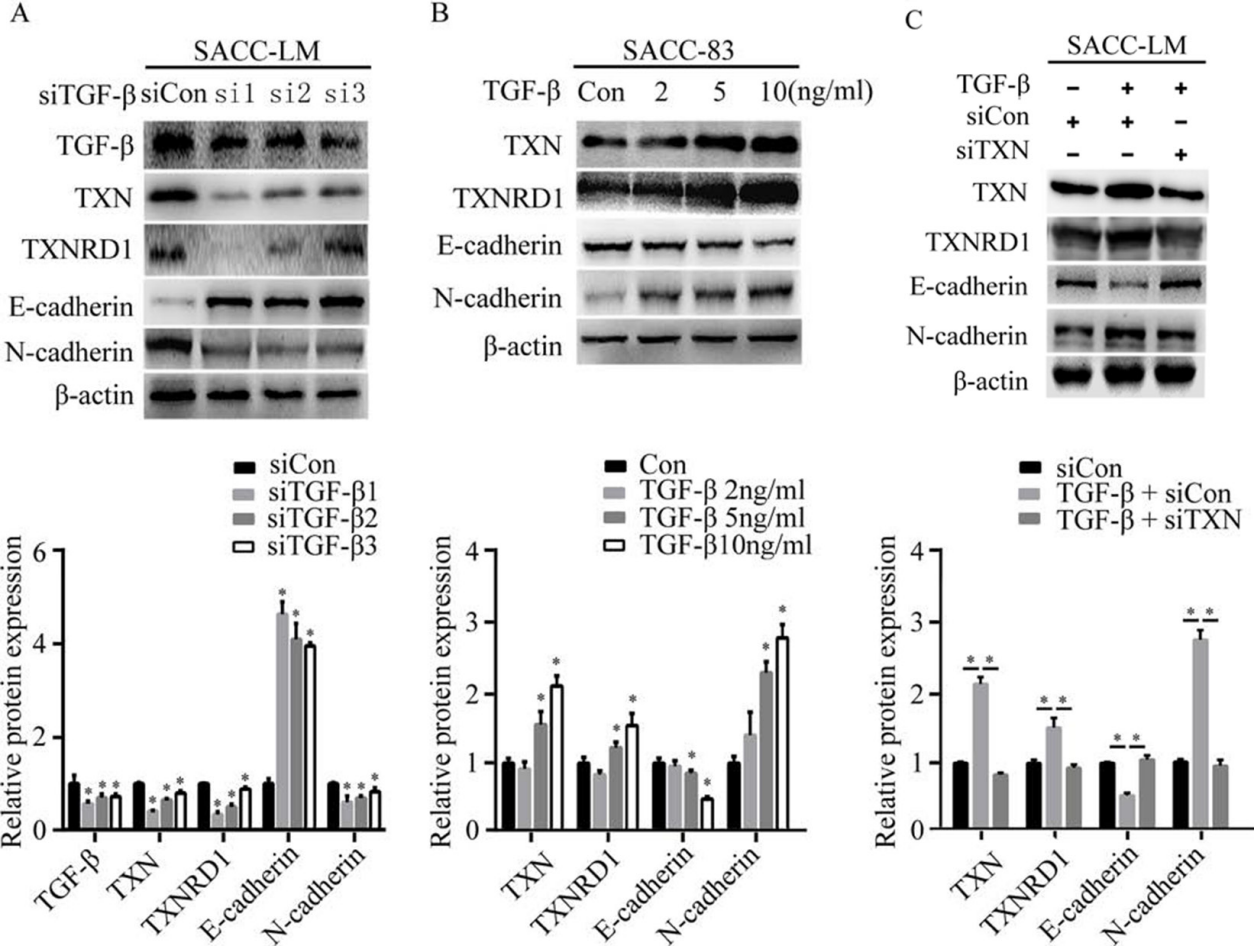
TXN is crucial for EMT induced by TGF-β in SACC cell lines **A.** Decreased TGF-β, TXN, TXNRD1, and N-cadherin expression and increased E-cadherin expression on immunoblots in TGF-β knockdown SACC-LM cells (si1, si2, si3) compared with control cells (siCon). **B.** Immunoblot analysis of expression of TXN, TXNRD1, E-cadherin, and N-cadherin in SACC-83 cells treated with or without TGF-β (2, 5 10 ng/ml) for 24 hours. **C.** After pre-transfection of TXN or Con siRNAs for 24 hours, siTXN or siCon cells were treated with or without TGF-β (10 ng/ml) for another 24 hours. Then expressions of TXN, TXNRD1, E-cadherin, and N-cadherin were analyzed. β-actin was used as loading control. Data represent means ± SD of three independent experiments (*P < 0.05).

SACC cells respond to TGF-β and acquire a mesenchymal phenotype through EMT, which is accompanied with a distinctive pattern of gene expression [9]. TGF-β stimulates EMT, migration, invasion, and metastasis of adenoid cystic carcinoma cells. To investigate how TGF-β affects TXN-dependent EMT in SACC cell lines, we knocked down TGF-β in SACC-LM by siRNA, and found that TXN protein expression and N-cadherin were downregulated, while E-cadherin were upregulated (Figure 4A). Treatment of SACC-83 with recombinant TGF-β induced TXN and N-cadherin expressions in a dose-dependent manner (Figure 4B), whereas TXN knockdown by siRNA suppressed effects of TGF-β on EMT (Figure 4C). These data suggest that TXN involves in the TGF-β-induced EMT in SACC cells.

### TXN regulates EMT through Akt/GSK-3β/Snail signaling pathway

**Figure 5:**
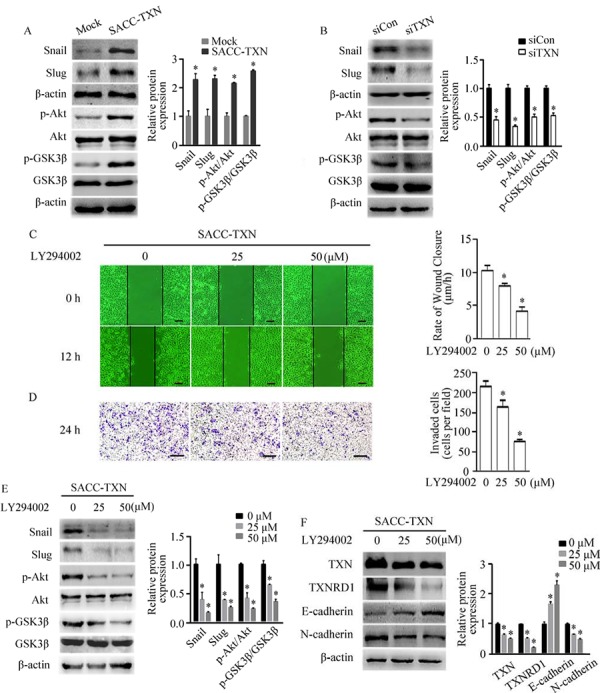
TXN regulates EMT in SACC cells through the Akt/GSK-3β/Snail signaling pathway Immunoblotting analysis of Snail, Slug, p-Akt/Akt, and p-GSK3β/GSK3β expression in Mock, SACC-TXN **A.** siCon, and siTXN **B.** cells. Wound closure assays (**C.** scale bar = 100 μm) and invasion assays (**D.** scale bar = 200 μm) in SACC-TXN treated with or without PI3K inhibitor LY294002 (25 μM, 50 μM) for 12 hours. Effects of LY294002 on expressions of Snail, Slug, p-Akt/Akt, and p-GSK3β/GSK3β **E.** or TXN, TXNRD1, E-cadherin, and N-cadherin **F.** in SACC-TXN cells. β-actin was used as loading control. Data represent means ± SD of three independent experiments (*P < 0.05).

Previous studies have demonstrated that TGF-β regulates Snail expression through PI3K/Akt /GSK-3β signaling [23, 24]. To further elucidate the mechanism of TXN induced cell motility, we examined above signaling components which are known to regulate cell migration and invasion. We found that overexpression of TXN increased the levels of Snail, Slug and Akt/GSK-3β phosphorylation, whereas knockdown of TXN reduced the levels of Snail, Slug, Akt/GSK-3β phosphorylation (Figure 5A and 5B). LY49002, PI3K inhibitor, suppressed motility and invasion (Figure 5C and 5D), and increased Snail, Slug, Akt/GSK-3β phosphorylation, TXN, TXNRD1 and N-cadherin while increased E-cadherin of SACC-TXN cells in a dose-dependent manner (Figure 5E and 5F). These findings suggest that TXN and PI3K/Akt/GSK-3β signaling coordinately regulate EMT of SACC cells.

### TXNRD1 targets Akt/GSK-3β to regulate EMT of SACC cells

**Figure 6:**
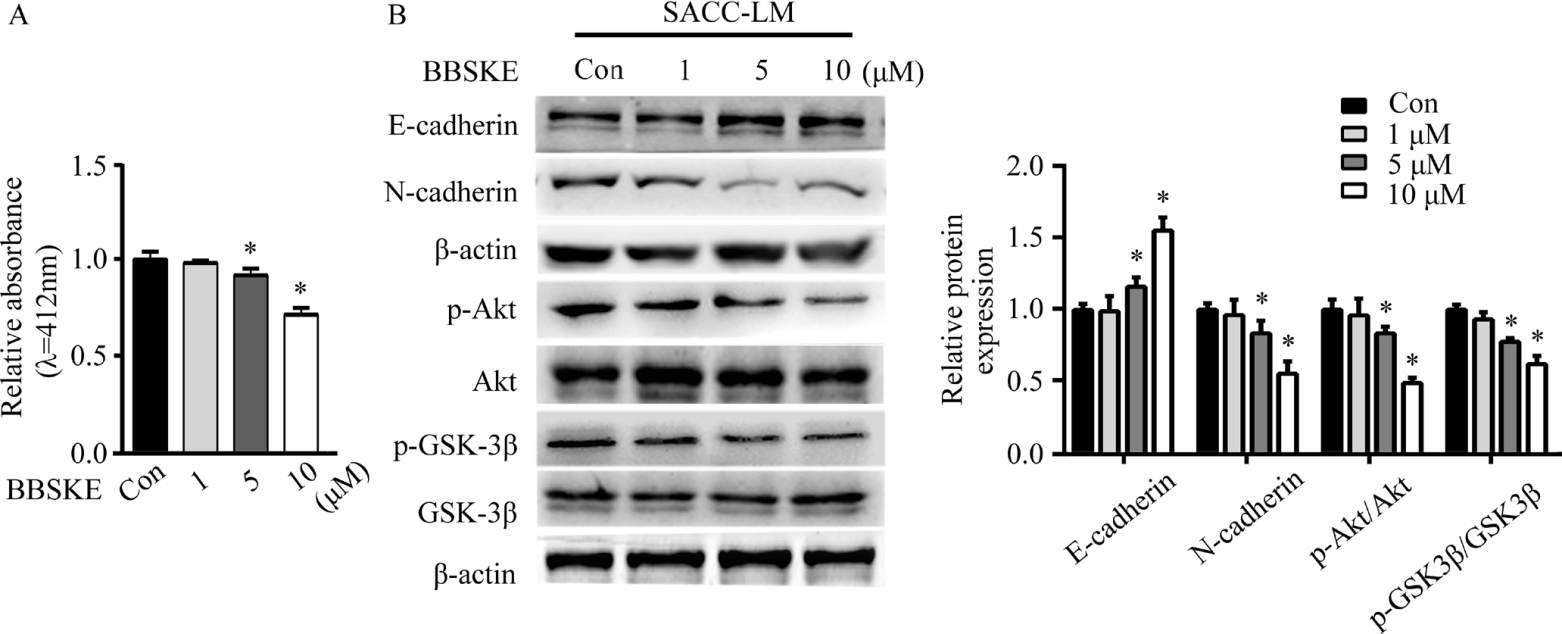
TXNRD1 inhibitor BBSKE inhibits EMT in SACC cells through the Akt/GSK-3β signaling pathway **A.** Effect of BBSKE on TXNRD1 activity in SACC-LM cells. TXNRD1 activity was measured of treatment with 1 μM, 5 μM, and 10 μM BBSKE after 48 h by DTNB assay. **B.** Immunoblotting analysis of E-cadherin, N-cadherin, p-Akt/Akt, and p-GSK3β/GSK-3β expressions in SACC-LM cells treated with BBSKE (1 μM, 5 μM, or 10 μM) for 24 hours. β-actin was used as loading control. Data represent means ± SD of three independent experiments (*P < 0.05).

**Figure 7:**
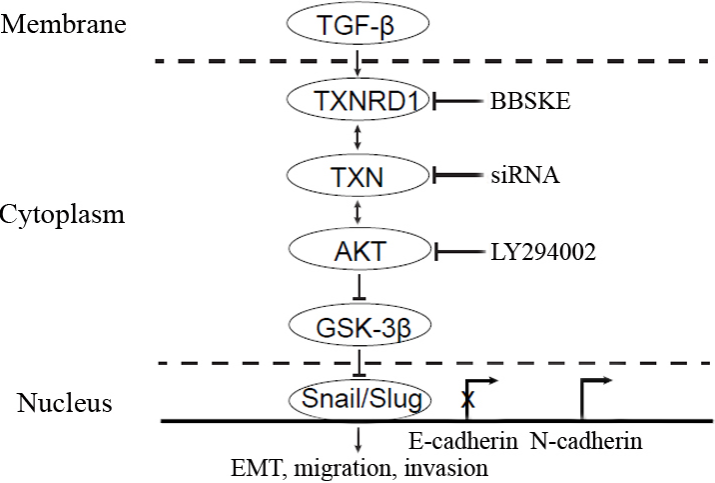
A hypothetical model demonstrating the essential roles of TXN in mediation of TGF-β induced EMT and SACC metastasis TXN in the cytoplasm mediated TGF-β induced EMT through Akt/GSK-3β signaling pathway, which could be blocked by TXNRD1 inhibitor (BBSKE) and siTXN. Overexpression of TXN leads to activation of Akt induced inactivation (phosphorylation) of GSK-3β, and repression of E-cadherin expression and increase of N-cadherin expression by transcription factor Snail and Slug. LY294002 could suppress Akt/GSK-3β, Snail and Slug which are the downstream signaling pathways of TXN in TXN-overexpressing cells.

TXNRD1 plays an important role in catalyzing the NADPH dependent regulation of TXN [25]. TXNRD1 knockdown markedly suppresses tumor progression and metastasis and decreases transcription levels of cancer-related proteins [26]. To examine the role of TXNRD1 in EMT of SACC-LM cells, we used TXNRD1 inhibitor, 1, 2- [bis (1, 2-Benzisoselenazolone-3 (2H) -ketone)] ethane (BBSKE) to treat SACC-LM cells and found that activity of TXNRD1 was decreased in a dose-dependent manner (Figure 6A). In addition, N-cadherin expression and Akt/GSK-3β phosphorylation were significantly decreased while E-cadherin expression was increased after BBSKE treatment in SACC-LM cells (Figure 6B). These findings imply that TXN and TXNRD1 could target PI3K/Akt/GSK3β which may impact EMT of SACC cells (Figure 7).

### TXN overexpression in SACC cells increases lung metastasis *in vivo*

**Figure 8:**
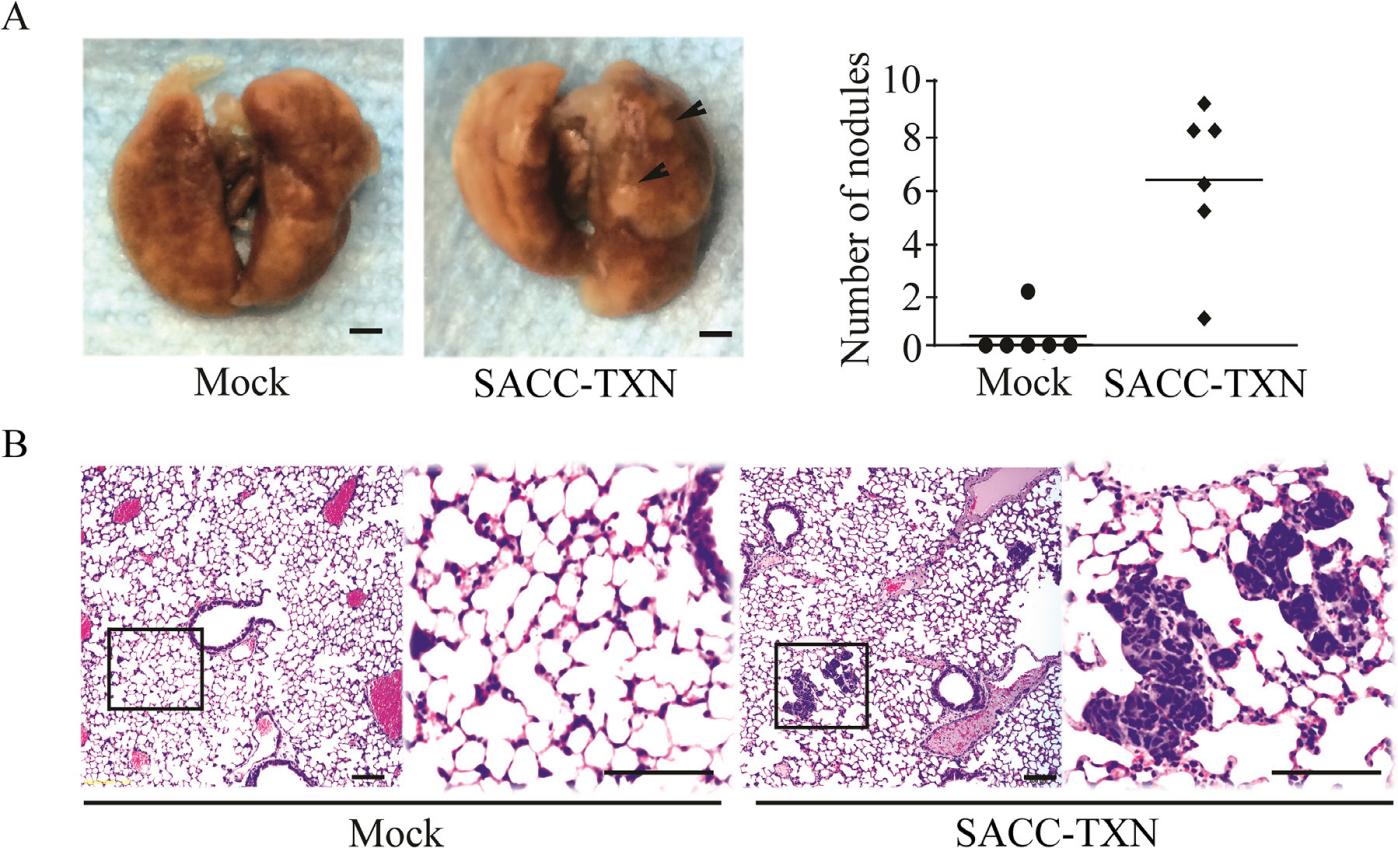
Overexpression of TXN enhances lung metastasis of SACC-83 cells *in vivo* **A.** Mice were inoculated intravenously with 3 × 10^6^ Mock or SACC-TXN cells. Eight weeks later, lung surface metastatic nodules (arrows) were observed and quantified. **B.** Representative hematoxylin and eosin staining showed that metastatic tumor cells in lung tissues section of mice injected intravenously with Mock or SACC-TXN cells. Scale bar = 100 μm.

To investigate the role of TXN in invasiveness and metastatic potential of SACC cells *in vivo*, 3 × 10^6^ SACC-TXN or Mock cells were injected into the tail vein of NOD/SCID mice. Eight weeks later, 83% mice injected with SACC-TXN cells developed lung metastases, compared to 17% mice injected with Mock cells (Figure 8A). Lung tissues of mice in SACC-TXN group had more tumor cells compared with those of mice in Mock group (Figure 8B). These results suggest that TXN might enhance SACC tumor cell metastasis *in vivo*.

## DISCUSSION

Previous studies suggest that both TXN and TXNRD1 are overexpressed in aggressive tumors with a high proliferation capacity, a low apoptosis rate, and an elevated metastatic potential [27]. In this study, we found higher expressions of TXN and TXNRD1 in the SACC tissue samples with distant metastasis. We also found that overexpression of TXN could increase lung metastasis of the SACC cells in a xenograft mouse model. Overexpression of TXN promoted an EMT-like phenotype with increased N-cadherin expression, enhanced cell mobility and invasion, and induced Snail/Slug expression through PI3K/Akt/GSK3β signaling pathway. These findings suggest that TXN and its related signaling pathway could be novel targets for therapeutic treatment of SACC.

TXN increases proliferation and resistance to cell death, and promotes metastatic progression of tumor cells [28]. It is predominantly located in cytoplasm, but also in the nucleus [29]. Nuclear translocation of TXN activates transcriptional factors involved in cellular redox regulation [30] and impacts high infiltration and/or metastatic capability of cancer cells [31]. Our present study found the occasional nuclear staining of TXN and TXNRD1 in SACC tumors with distant metastasis. In addition, increased expressions of TXN and TXNRD1 were correlated with clinical stage and distant metastasis in SACC patients, which supports previous report that TXN overexpression is an independent prognostic factor in metastatic cancers [30]. SACC-LM cells with high potential of metastasis had a higher expression of TXN compared with SACC-83 cells which have low potential of metastasis. In addition, when the SACC-83 cells were overexpressed TXN, they got an increase in EMT, cell migration and invasion. Combining with the role of EMT in chemoresistance and cancer cell invasion/metastasis [32], our data suggest that TXN may play a crucial role in regulation of SACC metastasis through EMT.

Our previous studies have shown that TGF-β induces EMT progression in SACC. In this study, we have further identified the role of TXN in TGF-β-induced EMT. Either knocking down TGF-β with siRNA in SACC-LM cells, or treating with TGF-β at different doses in SACC-83 cells, we found that TXN, TXNRD1, E-cadherin, and N-cadherin were regulated by TGF-β accordingly. In addition, knockdown of TXN followed by treatment with TGF-β could attenuate the effects of TGF-β on EMT. These data indicate that TXN is involved in the TGF-β-induced EMT in SACC.

Snail, a member of the zinc-finger transcription factor family, is a master regulator that promotes EMT and mediates invasiveness and metastasis in many different types of malignant tumors [33–35]. Snail and Slug are recognized as essential factors in EMT of SACC cells [36, 37]. In particular, Ras-MAPK activates Snail and Slug, which transcriptionally inhibit E-cadherin and promote EMT [38, 39]. Previous studies found that Snail was aberrantly expressed and essential for TGF-β-induced EMT in SACC [36, 40]. In this study, both Snail and Slug were upregulated in SACC-TXN cells. In addition, knockdown of TXN in SACC-LM cells inhibited Snail and Slug expressions. However, it is still unclear how Snail and Slug affect EMT in TXN overexpression SACC cells. Multiple signalings including Ras/MAPK, p38MAPK, Rho kinase, PI3-kinase, and Smad are related to the TGF-β-induced EMT [41]. The Akt/GSK-3β signaling pathway can modulate stability of Snail [24, 42]. PI3K/Akt pathway is also involved in cell migration, invasion and cell survival [43]. Our previous study have found that PI3K inhibitor LY294002 suppressed the EMT as well as decreased the migration ability of SACC cells [44]. TXN binds to and inhibits pro-apoptotic proteins, including apoptosis signal regulating kinase-1 (Ask-1) [45] and tumor suppressor PTEN [46], a protein that attenuates the PI3K/Akt cell survival pathway when it is present in cancer cells. Our data suggested that TXN might contribute to EMT modulated by PI3K/Akt /GSK-3β. It is supported by our findings that LY294002 suppressed expression of TXN and TXNRD1, while overexpression of TXN in SACC-83 cells impacts on Akt/GSK-3β phosphorylation. Previous studies have showed that GSK-3β is inhibited by TGF-β through increased serine 9 phosphorylation and increased stabilization of Snail via the PI3K/AKT signaling network [47].

TXNRD1 associated with aggressive tumor growth and poor survival rate plays an important role in regulation of TXN [25]. We further used BBSKE, a TXNRD1 activity inhibitor and demonstrated that BBSKE decreased TXNRD1 activity of SACC-LM in a dose dependent manner and downregulated N-cadherin expression, and increased E-cadherin expression via PI3K/Akt/GSK3β signal. It suggests that BBSKE may be a potential targeting agent in SACC metastasis through Akt/GSK-3β signaling pathway.

In summary, our present study demonstrated that TXN mediates TGF-β-induced EMT and promotes SACC metastasis through stabilization of the transcriptional factors, Snail and Slug, and cooperation with the PI3K/Akt/GSK-3β signaling. Our results suggest that TXN may serve as a prognostic marker for predicting the risk of developing distant metastases in SACC. In addition, TXN and its related signaling pathway could be potential therapeutic targets for SACC.

## MATERIALS AND METHODS

### Reagents and antibodies

1, 2- [bis (1, 2-Benzisoselenazolone-3 (2H) -ketone)] ethane (BBSKE) was obtained from the Organoselenium Research Center at Peking University, China. Human recombinant TGF-β1 was purchased from Sino Biological Inc., Beijing, China. Antibodies used for Western blotting were anti-TXN, anti-TXNRD1 (Epitomics, USA); anti-p-Akt (Ser478), anti-Akt, anti-E-cadherin, anti-N-cadherin, anti-p-GSK-3β (Ser9), anti-GSK-3β, anti-Snail, anti-Slug and anti-Twist (Cell Signaling Technology, Danvers, MA, USA), and anti-β-actin (Santa Cruz, USA). LY294002 was purchased from Beyotime Biotechnology Corporation (Haimen, China). NADPH and DTNB [5, 5′- dithiobis (2-nitrobenzoic acid)] were purchased from Sigma (St. Louis, MO, USA).

### Cell culture and plasmids

SACC-83 and SACC-LM cells were cultured in RPMI-1640 (Gibco, USA) supplemented with 10% fetal bovine serum (FBS, Gibco), 100 U/ml penicillin and 100 U/ml streptomycin at 37°C in a humidified atmosphere containing 5% CO_2_.

The plasmids encoding short interfering RNA (siRNA) targeting human TXN (siTXN), TGF-β (siTGF-β), and non-targeting control (siCon) were obtained from Guangzhou RiboBio (Guangzhou, China). SACC-LM cells were transfected with Lipofectamine 2000 (Invitrogen, Carlsbad, CA, USA) for 72 hours, and expressions of TXN and TGF-β were measured by Western blotting. For stable cell line selection, SACC-83 cells were transfected with TXN expressing GV230 vector (SACC-TXN) or the control GV230 vector (Mock). After incubation in 37°C for 48 h, the cells were treated with 500 μg/ml of G418 (Sigma-Aldrich, St. Louis, MO, USA), and cells that were resistant to G418 were collected.

### *In vitro* wound closure assay

After overnight serum deprivation, confluent monolayers of cells were scratched with a 200 μl pipette tip to create wounded areas with width of 400–600 μm. Wounded monolayers were photographed at 0 and 9/12 hours after scratching. Wound closure was determined by the reduction in the width of wounded areas. Average rates of wound closure were calculated as reduced width/incubated time.

### Transwell invasion assay

Transwell invasion assays were performed in the transwell chambers with a polycarbonate membrane (Millipore, Bedford, MA, USA) coated with 20 μg extracellular matrix (ECM) gel (Sigma). Cells were serum starved overnight, and then seeded at 2 × 10^5^ cells/well in medium without serum in the upper chamber, but with 15% fetal bovine serum (FBS) in the lower chamber. 24 hours after incubation, cells that had invaded through the matrix gel into the lower chamber were fixed with 95% ethanol and stained with 1% crystal violet (Sigma). Cells on the upper surface of the membrane were wiped off. Membranes were photographed in 4 random fields and the number of cells counted by light microscopy. Every experiment was repeated independently at least three times.

### TXNRD1 activity assay

After 48 hours-incubation with BBSKE of different concentrations (1 μM, 5 μM, 10 μM), SACC-LM cells were washed with PBS twice and lysed in lysis buffer (25 mM Tris-HCl pH 7.4, 1% Triton X-100, 1% sodium deoxycholate, 0.1% SDS, 137 mM NaCl, 10 mM EDTA). According to the procedure of our previous study [48], TXNRD1 activity was assayed in the cell lysates and reaction mixture (100 mM potassium phosphate pH 7.4, 0.3 mM NADPH, 150 μM DTNB). The conversion of 5, 5′- dithiobis (2-nitrobenzoic acid) (DTNB) to 5′- thionitrobenzoic acid (TNB) was measured spectrophotometrically at 412 nm. Each experiment was repeated independently three times.

### Western blot analysis

The protein extracts (30 μg) derived from each sample were separated on 10–15% SDS-PAGE and electroblotted onto polyvinylidenefluoride membranes. Non-specific binding was blocked with 5% non-fat milk in TBS-T (20 mM Tris, 137 mM NaCl, 0.1% Tween-20, pH 7.4) for 1 hour at room temperature. Blocked membranes were incubated overnight at 4°C with antibodies specific for TXN (1:10, 000), TXNRD1 (1:10, 000), p-Akt (1:2, 000), Akt (1:1, 000), p-GSK-3β (Ser9) (1:1, 000), GSK-3β (1:1, 000), E-cadherin (1:1, 000), N-cadherin (1:1, 000), Snail (1:1, 000), Slug (1:1, 000) and β-actin (1:1000), respectively. The membranes were then probed with HRP-conjugated secondary antibodies (1:4, 000–8, 000) at room temperature for 1 hour. Immunoreactive protein bands were visualized using the Fusion FX5 system (Vilber Lourmat, France). Quantity One software (Bio-Rad, USA) was used to analyze the densitometry data. Each experiment was repeated independently three times.

### Experimental metastasis assay

Five-week-old NOD/SCID female mice were purchased from Vital River Laboratories (Beijing, China). Mice were randomly grouped (6 mice per group). Mice were tail-vein injected with 3 × 10^6^ SACC-TXN or Mock cells in 100 μl PBS. After 8 weeks, mice were sacrificed. Lungs from mice were removed, fixed in 10% buffered formalin for 24 hours, examined with a microscope and scored for visible surface tumors. In addition, formalin fixed and paraffin embedded sections (4 μm) were used for Hematoxylin and Eosin staining. All animal care and procedures were approved by Peking University School of Stomatology Institutional Review Board for Animal Experiments.

### Immunohistochemistry

A cohort of 47 patients (20 male and 27 female with a median age of 46.5 years) diagnosed with SACC and treated at the Department of Oral and Maxillofacial Surgery, Peking University School of Stomatology during 1996–2006 were followed up. The mean follow-up period was 89.8 months. X-rays were performed to determine presence of pulmonary metastasis. Clinicopathological data were summarized in Table 1. Informed written consent was obtained from all subjects, and the study was approved by Peking University School of Stomatology Institutional Review Board.

SACC samples from these patients were formalin-fixed, paraffin-embedded, and sectioned (4 μm thick). Slides were deparaffinized in xylene for 2 hours and sequentially rehydrated through a graded ethanol series. Slides were incubated with 3% H_2_O_2_ for 30 minutes at room temperature, and then with 0.01 M citrate buffer (pH 6.0) for antigen retrieval. Immunostaining was performed by adding rabbit polyclonal TXN antibody (1:200, Sino Biological Inc., Beijing, China), rabbit polyclonal TXNRD1 antibody (1:200, Sino Biological Inc., Beijing, China), monoclonal mouse N-cadherin antibody (Santa Cruz, CA, USA, 1:200), or monoclonal mouse E-cadherin antibody (Santa Cruz, CA, USA, 1:200) overnight at 4°C. After incubation with 2-step plus Poly-HRP anti-mouse/rabbit IgG detection reagents (Zhongshan Golden Bridge Biological Technology Co., Ltd, Beijing, China) for 1 hour at room temperature, the slides were exposed to DAB (Zhongshan Golden Bridge Biological Technology Co., Ltd.), lightly counterstained with Mayer's hematoxylin, and covered with a thin glass coverslip.

For each primary tumor tissue section, ten random microscopic fields at a 100 × magnification were selected and positively stained cells were counted. Number of positively stained cells in each field was counted, averaged and scored as 0, 1, 2, or 3. 1) Score 0: no staining; 2) Score 1: weak staining (< 10% stained cells); 3) Score 2: moderate staining (10–90% stained cells); 4) Score 3: strong/intensive staining (> 90% stained cells). We defined cases with Score 0 and Score 1 as low expression, and Score 3 and Score 4 as high expression. Representative immunohistochemistry (IHC) score of TXN were shown in Figure S2.

### Statistical analysis

Data were presented as means ± SD. Statistical analysis was performed using SPSS Version 19.0 software. Data were grouped according to treatments and analyzed by one-way ANOVA. χ^2^ test was used to determine the correlation between TXN expression and clinicopathologic characteristics as well as TXNRD1, E-cadherin, and N-cadherin expressions. Overall survival rate was estimated using Kaplan-Meier method. *P* < 0.05 was considered statistically significant.

## SUPPLEMENTARY FIGURES AND TABLE



## References

[1] Bell D, Hanna EY (2013). Head and neck adenoid cystic carcinoma: what is new in biological markers and treatment?. Current Opinion in Otolaryngology & Head and Neck Surgery.

[2] Rapidis AD, Givalos N, Gakiopoulou H, Faratzis G, Stavrianos SD, Vilos GA, Douzinas EE, Patsouris E (2005). Adenoid cystic carcinoma of the head and neck. Clinicopathological analysis of 23 patients and review of the literature. Oral Oncol.

[3] Kokemueller H, Eckardt A, Brachvogel P, Hausamen JE (2004). Adenoid cystic carcinoma of the head and neck - a 20 years experience. International Journal of Oral and Maxillofacial Surgery.

[4] Liu J, Shao CB, Tan ML, Mu D, Ferris RL, Ha PK (2012). Molecular biology of adenoid cystic carcinoma. Head and Neck-Journal for the Sciences and Specialties of the Head and Neck.

[5] Kang Y, Massague J (2004). Epithelial-mesenchymal transitions: twist in development and metastasis. Cell.

[6] Thiery JP, Acloque H, Huang RY, Nieto MA (2009). Epithelial-mesenchymal transitions in development and disease. Cell.

[7] Ikushima H, Miyazono K (2010). TGFbeta signalling: a complex web in cancer progression. Nat Rev Cancer.

[8] Dong L, Ge XY, Wang YX, Yang LQ, Li SL, Yu GY, Gao Y, Fu J (2013). Transforming growth factor-beta and epithelial-mesenchymal transition are associated with pulmonary metastasis in adenoid cystic carcinoma. Oral Oncol.

[9] Dong L, Wang YX, Li SL, Yu GY, Gan YH, Li D, Wang CY (2011). TGF-beta1 promotes migration and invasion of salivary adenoid cystic carcinoma. J Dent Res.

[10] Lillig CH, Holmgren A (2007). Thioredoxin and related molecules—from biology to health and disease. Antioxid Redox Signal.

[11] Farina AR, Tacconelli A, Cappabianca L, Masciulli MP, Holmgren A, Beckett GJ, Gulino A, Mackay AR (2001). Thioredoxin alters the matrix metalloproteinase/tissue inhibitors of metalloproteinase balance and stimulates human SK-N-SH neuroblastoma cell invasion. Eur J Biochem.

[12] Mukherjee A, Martin SG (2008). The thioredoxin system: a key target in tumour and endothelial cells. Br J Radiol.

[13] Berggren M, Gallegos A, Gasdaska JR, Gasdaska PY, Warneke J, Powis G (1996). Thioredoxin and thioredoxin reductase gene expression in human tumors and cell lines, and the effects of serum stimulation and hypoxia. Anticancer Res.

[14] Fujii S, Nanbu Y, Nonogaki H, Konishi I, Mori T, Masutani H, Yodoi J (1991). Coexpression of adult T-cell leukemia-derived factor, a human thioredoxin homologue, and human papillomavirus DNA in neoplastic cervical squamous epithelium. Cancer.

[15] Nakamura H, Bai J, Nishinaka Y, Ueda S, Sasada T, Ohshio G, Imamura M, Takabayashi A, Yamaoka Y, Yodoi J (2000). Expression of thioredoxin and glutaredoxin, redox-regulating proteins, in pancreatic cancer. Cancer Detect Prev.

[16] Nakamura H, Masutani H, Tagaya Y, Yamauchi A, Inamoto T, Nanbu Y, Fujii S, Ozawa K, Yodoi J (1992). Expression and growth-promoting effect of adult T-cell leukemia-derived factor. A human thioredoxin homologue in hepatocellular carcinoma. Cancer.

[17] Watanabe R, Nakamura H, Masutani H, Yodoi J (2010). Anti-oxidative, anti-cancer and anti-inflammatory actions by thioredoxin 1 and thioredoxin-binding protein-2. Pharmacology & Therapeutics.

[18] Boudreau HE, Casterline BW, Rada B, Korzeniowska A, Leto TL (2012). Nox4 involvement in TGF-beta and SMAD3-driven induction of the epithelial-to-mesenchymal transition and migration of breast epithelial cells. Free Radic Biol Med.

[19] Cucoranu I, Clempus R, Dikalova A, Phelan PJ, Ariyan S, Dikalov S, Sorescu D (2005). NAD(P)H oxidase 4 mediates transforming growth factor-beta1-induced differentiation of cardiac fibroblasts into myofibroblasts. Circ Res.

[20] Rhyu DY, Yang Y, Ha H, Lee GT, Song JS, Uh ST, Lee HB (2005). Role of reactive oxygen species in TGF-beta1-induced mitogen-activated protein kinase activation and epithelial-mesenchymal transition in renal tubular epithelial cells. Journal of the American Society of Nephrology.

[21] Sturrock A, Cahill B, Norman K, Huecksteadt TP, Hill K, Sanders K, Karwande SV, Stringham JC, Bull DA, Gleich M, Kennedy TP, Hoidal JR (2006). Transforming growth factor-beta1 induces Nox4 NAD(P)H oxidase and reactive oxygen species-dependent proliferation in human pulmonary artery smooth muscle cells. Am J Physiol Lung Cell Mol Physiol.

[22] Masaki S, Masutani H, Yoshihara E, Yodoi J (2012). Deficiency of thioredoxin binding protein-2 (TBP-2) enhances TGF-beta signaling and promotes epithelial to mesenchymal transition. PLoS One.

[23] Chen KC, Chen CY, Lin CR, Yang TY, Chen TH, Wu LC, Wu CC (2013). Luteolin attenuates TGF-beta1-induced epithelial-mesenchymal transition of lung cancer cells by interfering in the PI3K/Akt-NF-kappaB-Snail pathway. Life Sci.

[24] Wang H, Fang R, Wang XF, Zhang F, Chen DY, Zhou BH, Wang HS, Cai SH, Du J (2013). Stabilization of Snail through AKT/GSK-3 beta signaling pathway is required for TNF-alpha-induced epithelial-mesenchymal transition in prostate cancer PC3 cells. European Journal of Pharmacology.

[25] Li C, Peng Y, Mao B, Qian K (2015). Thioredoxin reductase: A novel, independent prognostic marker in patients with hepatocellular carcinoma. Oncotarget.

[26] Yoo MH, Xu XM, Carlson BA, Gladyshev VN, Hatfield DL (2006). Thioredoxin reductase 1 deficiency reverses tumor phenotype and tumorigenicity of lung carcinoma cells. J Biol Chem.

[27] Lincoln DT, Ali Emadi EM, Tonissen KF, Clarke FM (2003). The thioredoxin-thioredoxin reductase system: over-expression in human cancer. Anticancer Res.

[28] Powis G, Kirkpatrick DL (2007). Thioredoxin signaling as a target for cancer therapy. Current Opinion in Pharmacology.

[29] Wei SJ, Botero A, Hirota K, Bradbury CM, Markovina S, Laszlo A, Spitz DR, Goswami PC, Yodoi J, Gius D (2000). Thioredoxin nuclear translocation and interaction with redox factor-1 activates the activator protein-1 transcription factor in response to ionizing radiation. Cancer Research.

[30] Noike T, Miwa S, Soeda J, Kobayashi A, Miyagawa S (2008). Increased expression of thioredoxin-1, vascular endothelial growth factor, and redox factor-1 is associated with poor prognosis in patients with liver metastasis from colorectal cancer. Hum Pathol.

[31] Nagano M, Hatakeyama K, Kai M, Nakamura H, Yodoi J, Asada Y, Chijiiwa K (2012). Nuclear expression of thioredoxin-1 in the invasion front is associated with outcome in patients with gallbladder carcinoma. Hpb.

[32] Maseki S, Ijichi K, Tanaka H, Fujii M, Hasegawa Y, Ogawa T, Murakami S, Kondo E, Nakanishi H (2012). Acquisition of EMT phenotype in the gefitinib-resistant cells of a head and neck squamous cell carcinoma cell line through Akt/GSK-3beta/snail signalling pathway. Br J Cancer.

[33] Naber HP, Drabsch Y, Snaar-Jagalska BE, ten Dijke P, van Laar T (2013). Snail and Slug, key regulators of TGF-beta-induced EMT, are sufficient for the induction of single-cell invasion. Biochem Biophys Res Commun.

[34] Wang Y, Shi J, Chai K, Ying X, Zhou BP (2013). The Role of Snail in EMT and Tumorigenesis. Curr Cancer Drug Targets.

[35] Wang D, Lu P, Zhang H, Luo M, Zhang X, Wei X, Gao J, Zhao Z, Liu C (2014). Oct-4 and Nanog promote the epithelial-mesenchymal transition of breast cancer stem cells and are associated with poor prognosis in breast cancer patients. Oncotarget.

[36] Shimoda M, Sugiura T, Imajyo I, Ishii K, Chigita S, Seki K, Kobayashi Y, Shirasuna K (2012). The T-box transcription factor Brachyury regulates epithelial-mesenchymal transition in association with cancer stem-like cells in adenoid cystic carcinoma cells. BMC Cancer.

[37] Tang Y, Liang X, Zheng M, Zhu Z, Zhu G, Yang J, Chen Y (2010). Expression of c-kit and Slug correlates with invasion and metastasis of salivary adenoid cystic carcinoma. Oral Oncol.

[38] Batlle E, Sancho E, Franci C, Dominguez D, Monfar M, Baulida J, de Herreros AG (2000). The transcription factor Snail is a repressor of E-cadherin gene expression in epithelial tumour cells. Nature Cell Biology.

[39] Cano A, Perez-Moreno MA, Rodrigo I, Locascio A, Blanco MJ, del Barrio MG, Portillo F, Nieto MA (2000). The transcription factor snail controls epithelial-mesenchymal transitions by repressing E-cadherin expression. Nature Cell Biology.

[40] Papagerakis S, Pannone G, Ogbureke KUE (2012). Epithelial-mesenchymal interactions in oral cancer metastasis. Oral Cancer.

[41] Kalluri R, Weinberg RA (2009). The basics of epithelial-mesenchymal transition. J Clin Invest.

[42] Wu Y, Deng J, Rychahou PG, Qiu SM, Evers BM, Zhou BPH (2009). Stabilization of Snail by NF-kappa B Is Required for Inflammation-Induced Cell Migration and Invasion. Cancer Cell.

[43] Toker A, Yoeli-Lerner M (2006). Akt signaling and cancer: surviving but not moving on. Cancer Research.

[44] Jiang Y, Ge XY, Liu SM, Zheng L, Huang MW, Shi Y, Fu J, Zhang JG, Li SL (2014). Nimotuzumab suppresses epithelial-mesenchymal transition and enhances apoptosis in low-dose UV-C treated salivary adenoid cystic carcinoma cell lines *in vitro*. Anticancer Drugs.

[45] Saitoh M, Nishitoh H, Fujii M, Takeda K, Tobiume K, Sawada Y, Kawabata M, Miyazono K, Ichijo H (1998). Mammalian thioredoxin is a direct inhibitor of apoptosis signal-regulating kinase (ASK) 1. Embo Journal.

[46] Meuillet EJ, Mahadevan D, Berggren M, Coon A, Powis G (2004). Thioredoxin-1 binds to the C2 domain of PTEN inhibiting PTEN's lipid phosphatase activity and membrane binding: a mechanism for the functional loss of PTEN's tumor suppressor activity. Arch Biochem Biophys.

[47] Cho HJ, Baek KE, Saika S, Jeong MJ, Yoo J (2007). Snail is required for transforming growth factor-beta-induced epithelial-mesenchymal transition by activating PI3 kinase/Akt signal pathway. Biochemical and Biophysical Research Communications.

[48] Xing F, Li S, Ge X, Wang C, Zeng H, Li D, Dong L (2008). The inhibitory effect of a novel organoselenium compound BBSKE on the tongue cancer Tca8113 *in vitro* and *in vivo*. Oral Oncol.

