# Supplementary material for: Thioredoxin 1 mediates TGF-β-induced epithelial-mesenchymal transition in salivary adenoid cystic carcinoma
Source: Oncotarget. 2015 Aug 17;6(28):25506–19. doi: 10.18632/oncotarget.4635 (PMC4694848; doi:10.18632/oncotarget.4635)
Supplement: Supplementary file 1 [file oncotarget-06-25506-s001.pdf]

SUPPLEMENTARY FIGURES AND TABLE

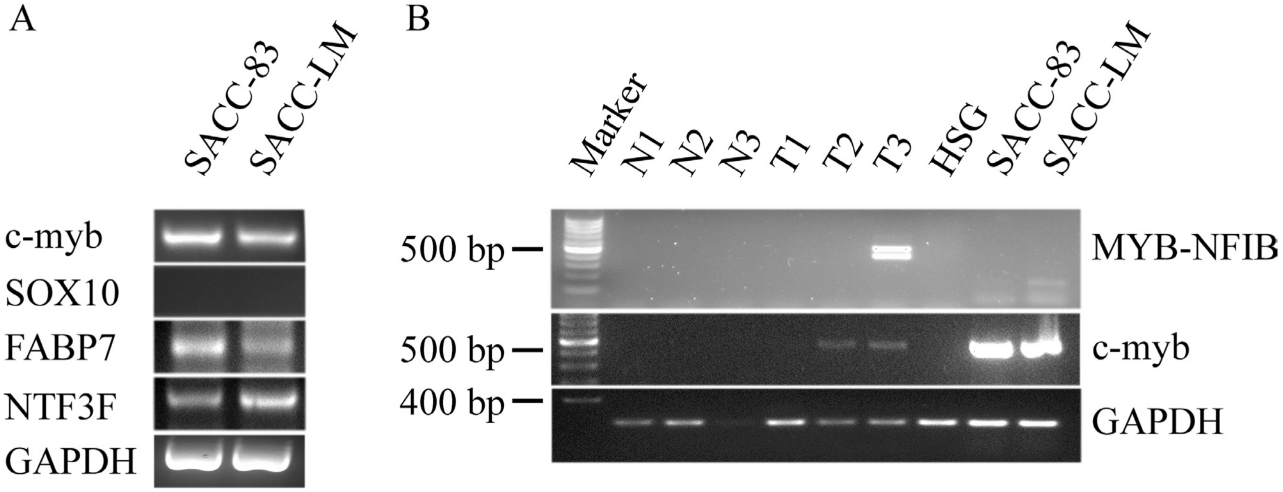

Supplementary Figure S1: Detection of intrinsic characteristics of SACC. A. RT-PCR analysis of intrinsic markers (c-myb, SOX10, FABP7, NTF3F) of SACC in SACC-83 and SACC-LM. **B.** MYB-NFIB fusion, c-myb, and GAPDH transcripts using the indicated primers in fresh frozen tissues of normal salivary glands (N1-N3), SACCs (T1-T3) and a human salivary gland (HSG) cell line, SACC-83 and SACC-LM cell lines by RT-PCR. GAPDH was used as internal control. Data represent three independent experiments.

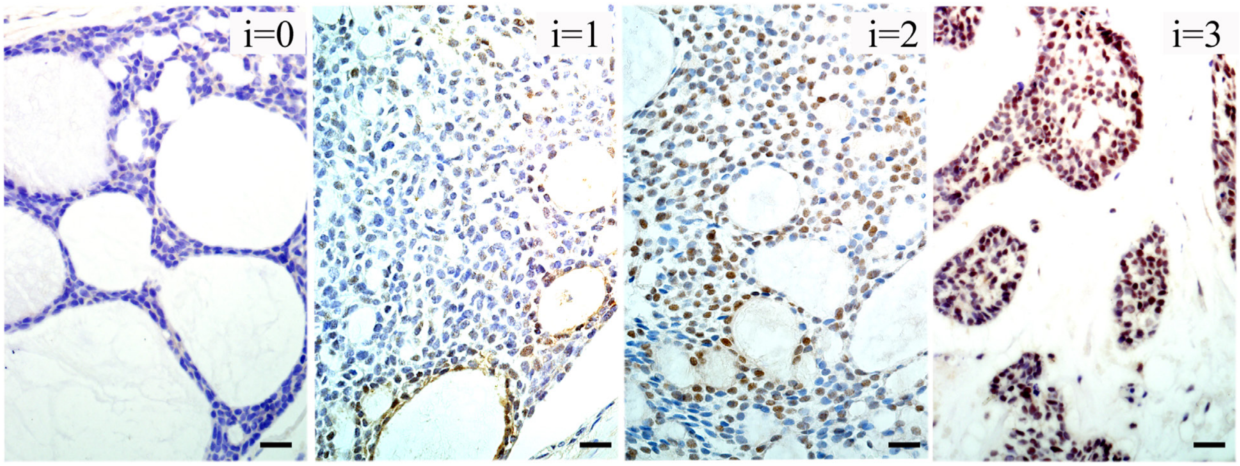

Supplementary Figure S2: The immunohistochemical criteria for TXN staining of SACCs. Different immunoreactivity scores for TXN staining in SACC tissues: i = 0 (no staining), 1 (< 10% stained cells), 2 (10–90% stained cells) and 3 (> 90% stained cells); bar = 20 μm.

Supplementary Table S1: Primer sequences used to detect mRNA expression

Primer name	Primer sequence
c-myb	AATTAAATACGGTCCCCTGAA
	TGCTCCTCCATCTTTCCACAG
SOX10	GGTAATGTCCAACATGGAGACC
	GTAGGCGATCTGTGAGGTGG
FABP7	AAGTCTGTTGTTAGCCTGGA
	CTGCCTCCACACCAAAGACA
NTF3F	GGGCCCGCCAAGTCAGCATT
	TATCCACCGCCAGCCCACG
MYB-1693 F 12	GCAGGATGTGATCAAACAGG
NFIB-1197 R 9	CCGGTAAGATGGGTGTCCTA
GAPDH	CAATTCCGAGAGCGTTCC
	CAAAGTTGTCATGGATGACC
